# 590 An Occupation-Based Review of Outcome Assessments Used in Hand Function After Burn Injury

**DOI:** 10.1093/jbcr/irae036.224

**Published:** 2024-04-17

**Authors:** Jill M Cancio, Wendy Stav

**Affiliations:** US Army Institute of Surgical Research, Floresville, TX; Saint Louis University, St. Louis, MO; US Army Institute of Surgical Research, Floresville, TX; Saint Louis University, St. Louis, MO

## Abstract

**Introduction:**

Since over 50% of those who survive burn injury have hand involvement, one of the most important functional outcomes of burn rehabilitation is hand function. Occupational therapists leverage the use of occupation to restore function. While occupation began as the central concept of occupational therapy at the dawn of the profession, the use of occupation throughout the therapy process, including assessment, has wavered in recent decades. Knowledge of the occupational nature of assessments is important to the delivery of authentic occupational therapy.

**Methods:**

Outcome measures that have been cited in the burn rehabilitation literature to assess hand function were included in this review and were classified into 3 categories (traditional components, performance, and patient-reported outcome measures). Psychometrics to include established validity in the burn population were also described. The extent to which each measure utilizes occupation to assess function was determined with the Occupation Based Performance Assessment (OPBA). The OBPA consists of several scales (authentic occupation, meaningful and purposeful value, and therapeutic intent) to measure the presence of occupation in assessment tools and practice patterns used in occupational therapy practice. The OPBA is interpreted along a continuum divided into 3 categories: occupation-based, intermediate, or discrete.

**Results:**

The searches of the data bases yielded identification of 2 traditional component measures, 4 performance measures, and 4 patient-reported measures. According to the OPBA there are 3 discrete measures, 6 intermediate measures, and 1 occupation-based measure. Of the 10 assessments identified in the literature, 6 are performance-based assessment tools. None of the performance-based assessments were classified above intermediate on the occupation-based continuum. A novel occupation-based measure was identified, the Suitcase Packing Activity (SPA); however, it has not been validated in the burn population.

**Conclusions:**

This review of outcome measures to assess hand function after burn injury revealed a paucity of occupation-based assessments in this practice area which has implications for delivery of occupation-based care. The majority of outcome measures used in upper extremity burn rehabilitation are discrete in nature and do not measure occupations of clients. The SPA, after validation in the burn population, may be a viable addition to an assessment battery to address this essential need.

**Applicability of Research to Practice:**

There is a paucity of occupation-based assessments in this practice area which has implications for delivery of occupation-based care. Valid occupation-based performance measures in the burn population are needed.

